# Experiences of integrating social prescribing link workers into primary care in England — bolting on, fitting in, or belonging: a realist evaluation

**DOI:** 10.3399/BJGP.2024.0279

**Published:** 2025-01-28

**Authors:** Stephanie Tierney, Debra Westlake, Geoffrey Wong, Amadea Turk, Steven Markham, Jordan Gorenberg, Joanne Reeve, Caroline Mitchell, Kerryn Husk, Sabi Redwood, Catherine Pope, Beccy Baird, Kamal Ram Mahtani

**Affiliations:** Nuffield Department of Primary Care Health Sciences, University of Oxford, Oxford.; Nuffield Department of Primary Care Health Sciences, University of Oxford, Oxford.; Nuffield Department of Primary Care Health Sciences, University of Oxford, Oxford.; Nuffield Department of Primary Care Health Sciences, University of Oxford, Oxford.; Nuffield Department of Primary Care Health Sciences, University of Oxford, Oxford.; Nuffield Department of Primary Care Health Sciences, University of Oxford, Oxford.; Hull York Medical School, University of Hull, Hull.; School of Medicine and Population Health, University of Sheffield, Sheffield.; Peninsula Medical School, University of Plymouth, Plymouth.; Bristol Medical School, University of Bristol, Bristol.; Nuffield Department of Primary Care Health Sciences, University of Oxford, Oxford.; The King’s Fund, London.; Nuffield Department of Primary Care Health Sciences, University of Oxford, Oxford.

**Keywords:** link workers, primary health care, realist evaluation, social prescribing, delivery of health care

## Abstract

**Background:**

Following the 2019 NHS Long Term Plan, link workers have been employed across primary care in England to deliver social prescribing.

**Aim:**

To understand and explain how the link worker role is being implemented in primary care in England.

**Design and setting:**

This was a realist evaluation undertaken in England, focusing on link workers based in primary care.

**Method:**

The study used focused ethnographies around seven link workers from different parts of England. As part of this, we interviewed 61 patients and 93 professionals from health care and the voluntary, community, and social enterprise sector. We reinterviewed 41 patients, seven link workers, and a link worker manager 9–12 months after their first interview.

**Results:**

We developed four concepts from the codes developed during the project on the topic around how link workers are integrated (or not) within primary care: (or not) within primary care: centralising or diffusing power; forging an identity in general practice; demonstrating effect; and building a facilitative infrastructure. These concepts informed the development of a programme theory around a continuum of integration of link workers into primary care — from being ‘bolted on’ to existing provision, without much consideration, to ‘fitting in’, shaping what is delivered to be accommodating, through to ‘belonging’, whereby they are accepted as a legitimate source of support, making a valued contribution to patients’ broader wellbeing.

**Conclusion:**

Social prescribing was introduced into primary care to promote greater attention to the full range of factors affecting patients’ health and wellbeing, beyond biomedicine. For that to happen, our analysis highlights the need for a whole-system approach to defining, delivering, and maintaining this new part of practice.

## Introduction

Patients often present to GPs with health problems that stem from or that are aggravated by non-medical factors (for example, inadequate housing, financial issues, bereavement, and/or loneliness); this is estimated to apply to one in five patients seen by a GP.^1^ Social prescribing has become an established means of addressing non-medical determinants of health in a number of countries,^2^^,^^3^ with England regarded as being at the forefront of its advancement.^4^ Social prescribing forms part of the comprehensive model of personalised care developed by the NHS in England.^5^ Personalised care aims to provide people with choice and control, focusing on what matters to them and drawing on individual strengths and needs.^6^

NHS England has provided funding for social prescribing link workers to serve primary care.^6^^,^^7^ Link workers identify non-medical factors contributing to a patient’s illness or poor wellbeing and, when appropriate, connect people to support or advice, often through the voluntary, community, and social enterprise (VCSE) sector. Despite this national roll out, social prescribing delivery in England varies. For example, link workers might be employed through primary care or subcontracted to a VCSE organisation,^8^ and there is variation in the frequency and duration of interactions with patients, the intensity of support offered (for example, accompanying people to groups and/or activities), and what access they have to primary care IT systems.^9^^,^^10^

Previous research^4^^,^^11^ has highlighted the complex and often conflicting nature of social prescribing delivery in England owing to:

- general practice taking an approach that is holistic (seeking to understand and collaborate) or fragmented (patients’ non-medical needs being seen as ‘outsourced’ to a link worker);
- link workers focusing on the relational (having open-ended interactions to respond to patients’ changing needs) or transactional (following a firm set of rules around how social prescribing is delivered); and
- the VCSE’s role being redistributive (valued and sustainable) or non-redistributive (a lack of services available to support those most in need).

**Table:** How this fits in

Social prescribing has been rolled out nationally in primary care in England; its delivery across the country varies. Our research shows how those developing, delivering, and funding social prescribing have to consider the infrastructure around link workers. This is important to ensure that these employees feel they belong in primary care rather than being bolted on to this setting. Practices can foster this sense of belonging by ensuring that link workers receive a comprehensive induction and are invited to team meetings, by developing clear information about what patients can expect from social prescribing, appreciating that the approach taken by link workers when supporting patients may require longer consultations than for medical complaints, and allowing link workers time in their working week to establish connections with local groups, organisations, and charities.

This complexity underpinned a previous realist review we conducted on link workers;^12^ it highlighted the importance of buy-in to this role, and to the person employed as a link worker, by patients, healthcare staff, and VCSE organisations. It noted that connections between link workers and these stakeholders were important for such buy-in, a finding noted in other studies.^11^

A follow-up realist evaluation was undertaken to advance ideas from our review.^12^ It involved focused ethnographies, an approach used in other studies on social prescribing in single parts of England.^13^^,^^14^ Our realist evaluation involved data collection from a range of geographical locations in England, which helped with understanding factors affecting how and/or if link workers become part of primary care — the focus of this article.

## Method

Details of the approach taken to data collection and analysis are reported in our protocol^15^ and elsewhere.^16^ A range of data (interviews, observations, documents, patient questionnaires, daily debriefs with link workers, and rates of GP usage for patients before and after referral to a link worker) were collected around seven link workers based across England. This article centres on data collected during interviews and observations (for example, link workers meeting with patients, with primary care staff, and with VCSE organisations).

Variation was sought among the link workers involved through purposive sampling — in terms of whether they were employed via primary care or a VCSE organisation, the part of the country they worked in and how deprived it was, how many GP practices they served, and length of time in the post. Between November 2021 and November 2022, researchers spent 3 weeks with each of the seven link workers, making fieldnotes of what they observed (which were typed into a Microsoft Word document for analysis). They also interviewed link workers, patients they supported, healthcare staff, and VCSE representatives. These interviews lasted between 20 and 65 minutes. Patients and link workers were reinterviewed 9–12 months later. These interviews lasted between 15 and 50 minutes. Interviews were conducted in person, by telephone, or video via Microsoft Teams, and were transcribed verbatim. Initially, interview topic guides were informed by our previous realist review,^12^ in consultation with the study’s patient and public involvement group and advisory group. It was amended as data collection proceeded to incorporate new understandings (for example, items added about discretion link workers had in their role).

Analysis was concurrent with data collection. Data coding was supported by using the qualitative software NVivo (version 12); three researchers were involved in this process. Interview data were coded first, followed by fieldnotes. Analysis involved initially developing a coding framework, informed by concepts from our earlier realist review;^12^ inductive codes were added when sections of data did not fit this. These codes were used to develop Context–Mechanism–Outcome Configurations (CMOCs) — a key part of realist evaluations that help to explain how programmes or interventions work, for whom, under what conditions, and why.^17^ Coded data were used to consider whether CMOCs from the review needed amending. New CMOCs were developed when necessary. A number of reasoning processes were employed (for example, juxtaposing data, unpicking conflicting data, and consolidating data) to explore and explain differences in outcomes.

A set of codes (see Box 1) was collated in NVivo12 under the heading ‘belonging’. We used these codes to identify outcomes on how far link workers were embedded into primary care (for example, link workers being bolted on, fitting in, or belonging) and considered what these data suggested in terms of mechanisms and context required to trigger them. CMOCs helped us develop a programme theory on embedding link workers into primary care, which is described below.

**Box 1.** Codes collated in NVivo under the concept of ‘belonging’

Appreciation of the link worker role Appropriateness of referrals Communication in primary care Credibility of the link worker role Demonstrating effect Fitting into medical systems Funding GP workload Investment in VCSE Number of referrals Openness to change Joint working across sectors Time to work with the VCSE Space to see patients Supervision Understanding of the role Uniqueness in primary care

*VCSE = voluntary, community, and social enterprise.*

## Results

We interviewed 93 professionals (VCSE staff, GPs, link workers, practice managers, nurses, care coordinators, health and wellbeing coaches, reception staff, and allied health professionals) and 61 patients. Details of these interviewees are listed in Tables 1 and 2. We reinterviewed 41 patients, seven link workers, and one link worker manager. Of the patients we interviewed, 12 took part in an observation of their meeting with a link worker. Another 23 patients took part in an observation but not an interview.

**Table 1.** Background information on professionals who were interviewed^a^

**Characteristic**	***n* ^b^**
**Work roles**	
Link workers^c^	12
VCSE staff and managers	20
GPs (including registrars)	19
Practice managers/operations managers	11
Nurses (including advanced practitioners)	10
Care coordinators/health and wellbeing coaches	6
Reception staff	5
Clinical pharmacists	2
Mental health practitioners	2
Dietitian	1
Occupational therapist	1
Paramedic	1
Physiotherapist	1
Other	2

**Gender**	
Female	70
Male	23

**Age, years**	
Range	20–66
Mean (SD)	43.3 (12.2)

a *One of the VCSE staff was a line manager to two of the link workers involved in the study, so was interviewed twice.*

b *Unless otherwise specified.*

c *Seven of these link workers acted as cases in the study; the five others were their colleagues. SD = standard deviation. VCSE = voluntary, community, and social enterprise.*

**Table 2.** Background information on participating patients (interviews and/or observations)^a^

**Characteristic**	***n* ^b^**
**Involvement in the study**	
Observation only (appointment between link worker and patient)	23
Interview only	49
Interview and observation	12

**Ethnicity**	
White British	62
White (non-British)	6
Asian (including British Asian and Indian)	5
African Caribbean/Black British	5
Mixed ethnic groups	3
Other	3

**Gender**	
Female	55
Male	29

**Age, years**	
Range	19–86
Mean (SD)	49.3 (19.5)

a *One interviewee was speaking as a member of the practice patient participation group rather than someone who had engaged in social prescribing.*

b *Unless otherwise specified. SD = standard deviation.*

Codes on belonging were clustered into four key concepts related to power, professional identity, establishing impact, and having a supportive infrastructure. These concepts are explored below.

### Centralising or diffusing power

The need to diversify support provided in primary care, and embrace and address the broader determinants of health, was accepted by many healthcare professionals we interviewed. However, for most sites, power related to the delivery of social prescribing resided in a primary care network (PCN), as the organisational unit that ultimately held the funding for link workers. This could be problematic if a PCN failed to consult with key stakeholders (for example, VCSE representatives and patients) when planning a social prescribing service; provision might then be developed to fit with existing ways of working:

*‘… there were five social prescribers over five surgeries … four … left consecutively over a 6 to 9 month period. All highly experienced … All brilliant at their jobs … they were being challenged … told off for things that they really weren’t responsible for. The back to front working like, “You need to do this but we’re not going to give you the time to do it, but if you’ve done that, why have you done that?”.’*

(Site 2, link worker [LW]02, follow-up interview)

Problems could transpire when link workers were funded through a PCN but employed by a VCSE organisation. In such cases, link workers could be uncertain where to expect supervision and guidance. Confusion arose about which organisation’s ‘rules’ (for example, protocols and procedures) to follow, putting link workers at risk of receiving insufficient support or guidance in their job:

*‘Pros and cons of being employed by a VCSE from talking to the link worker. Pros = you are a little bit protected — gives some cover when things are not going well — can say “my employer says that …” Cons = you feel you are not so embedded in the team … she served different surgeries, with different logins and systems and teams to make relationship with. No clear guidance about home working from the different surgeries … She feels she is caught between different organisations with different expectations and requirements.’*

(Site 1, researcher fieldnotes)

For social prescribing to be successful, data highlighted that the VCSE sector had to be able to work alongside health care; its contribution to social prescribing was regarded as essential but could be underplayed, underappreciated, and underfunded:

*‘… we constantly move people into another service which is equally, or often worse funded … if you’re moving them into the voluntary sector, they’re often very badly funded and overloaded … ’*

(Site 2, healthcare professional [HCP]06)

*‘…* [social prescribing] *is only as effective as the groups that you are connecting people to … if you’re feeling a bit rubbish anyway, you don’t want to go along to a group that’s in financial trouble and might end in a few weeks — imagine putting in all that effort, and then suddenly you can’t go anymore.’*

(Site 4, LW01)

Some disquiet was expressed within interviews around health care infringing on the VCSE’s ‘turf’, through social prescribing, without due consideration of the work this sector had undertaken previously to support people’s broader health. At the same time, smaller VCSE organisations felt that social prescribing made them accessible to a range of people, through link worker referrals:

*‘… you’ve got the larger established voluntary sector organisations who — for want of a better word — are trying to hold onto their funding … and they’re using their own resources and, actually, not fairly and inclusively distributing referrals to the right people … what I love about* [link workers] *… they really are so impartial … ’*

(Site 5, VCSE01)

### Forging an identity in general practice

Most link workers were new to working in a healthcare setting, and it could take time acclimatising to its culture and systems. They recalled entering the job with only a vague idea of what they were expected to do, becoming aware of what practices wanted once in post. A lack of professional qualification in social prescribing could be a barrier to link workers’ legitimacy in primary care, even though they generally came to the role with a range of relevant professional and sometimes personal experience (for example, of the local community and of difficult life circumstances). External policy could help to support their status:

*‘I think The Long Term Plan has given a lot more visibility to social prescribing. It’s giving it a lot more credibility in the eyes of the medical community … And supports us towards a broader objective of getting parity of esteem in terms of non-medical and medical interventions and what different partners can bring to that.’*

(Site 6, HCP01)

However, legitimacy of link workers appeared to be shaped, to a large degree, through the interpersonal interactions these employees had with GPs and other dominant actors in primary care:

*‘… she’ll* [link worker] *come and talk to me at the meeting and tell me a bit of an update about someone she’s spoken to. Or she’ll ask me something about one of my patients. Now that I know her I can understand the role more. Also, if you know somebody you feel happier referring to them … ’*

(Site 3, HCP09)

For link workers to become part of primary care, staff needed to understand their role; when this was lacking, staff questioned link workers’ actions, including the amount of time they spent with patients and the intensity of engagement (for example, going to community activities with people). This could create some resentment:

*‘… one of my patients wanted me to attend a group with them … it happened to be a relaxation group … the reception staff wanted to know where I was … people think social prescribers have it easy, they’re always doing activities … apparently this one particular receptionist … complained about me to my manager saying, “We couldn’t find her”. But it was in my diary … ’*

(Site 4, LW02, follow-up interview)

*‘… if you’re working 10 minutes and the person next door to you is doing 1 hour and two* [patients] *don’t turn up you can then start having that resentment of “Why are they here?” … then you feel they’re not a member of the team … ’*

(Site 1, HCP01)

Attempts to build understanding, and to foster link workers’ legitimacy in primary care, included giving presentations or providing opportunities for staff to shadow this workforce. Having a link worker located in primary care was said to help staff understand this role and to develop a rapport with the person delivering it, although this could be difficult if a link worker was part time and/or served several practices. Link workers’ physical presence in primary care reminded GPs about their contribution to patient care, and meant they could have informal conversations about whether a referral was appropriate:

*‘… we did have like a little paper, kind of referral form. You just went upstairs and you filled it in, but actually you’d have to walk past her* [link worker] *desk to fill it in and you’d have a little chat. You know I’ve got this person, what do you think, is it somebody that you think you might be able to work with? So that was really helpful … ’*

(Site 6, HCP08)

Some interviewees commented that patients were more receptive to social prescribing if it was associated with a GP practice. At the same time, patients may come to this space with preconceptions of what assistance they would receive, making the offer of non-medical support disorientating. This dissonance was heightened when link workers saw patients in a typical GP consultation room:

*‘I feel like doing that in a doctor’s surgery does feel a little bit clinical. And she was put into a room that was — it had a bed and everything so it did feel very doctor … It would be nice if she had more of a living room feel to her space.’*

(Site 2, patient [P]13)

In terms of patients’ understanding, some practices tried to increase awareness of the link worker role — sending out information in newsletters and having recorded messages that played when people rang to make an appointment. Yet the link worker role appeared to be unclear to many patients referred to them, as reflected in this interview excerpt:

*‘I haven’t got a scooby doo to be honest … how I ended up having that phone call is a complete mystery to me. I just went along with it. I thought it must be happening for a reason … I’m not quite sure what the sort of ultimate goal of it* [social prescribing] *is or what we’re sort of working towards exactly, but yes I mean I know, she’s* [link worker] *very easy to talk to … ’*

(Site 5, P04)

### Demonstrating effect

Some link workers described being judged on health systems process indicators of success — such as number of patients seen each day and throughput. However, it was suggested during interviews that social prescribing did not necessarily offer a short-term or immediate solution. Some interviewees noted that link workers provided a less transactional approach than medical staff, having time to disentangle the various non-medical layers that were part of a patient’s presenting problem. GPs stated in interviews that access to a link worker gave them another route for assisting patients, *‘spreading the load’* (Site 7, HCP07) within a practice. Some believed their caseload had reduced because of the provision of social prescribing, although this was not the experience of everyone:

*‘I’m definitely seeing less patients coming to me as a first port of call with the sort of problems which are very time-consuming from our point of view and may not have required my time, and by reducing that it does free up my time to do other things.’*

(Site 2, HCP06)

*‘… it’s really hard to quantify number of appointments saved and* [link worker] *will tell you that a lot of times she’ll encourage them to come to the GP. But that’s what you want if they have medical health issues … ’*

(Site 5, HCP01)

Link workers described observing signals that a patient’s situation had improved — someone showing greater confidence, leaving their house, or trying new things. GPs and link workers might also receive *ad hoc* positive feedback from individual patients:

*‘… for us it’s just patients coming back and saying they were pleased for the service. They did x, y, and z and things like that. I think the only way to clarify if it works would be actually if you could speak to patients … ’*

(Site 7, HCP01)

Link workers’ ability to demonstrate impact was compromised by a lack of system to report back to the referrer (usually a GP) about what had happened with a patient. In addition, it was uncommon for link workers to hear from VCSE organisations about how a patient had fared after being referred through social prescribing. It was proposed during interviews that resourcing and supporting more formal reporting across stakeholders could help to sustain the link worker role. When link workers did report back to GPs and other primary care staff, they often included information on how many people they had seen over a specific time period and summaries of patients’ stories. They might also include data recorded on questionnaires that patients completed (for example, on wellbeing). However, most link workers were against collecting data when doing so could interfere with their ability to connect with patients, when they thought this was for the benefit of others (for example, managers and funders) rather than the patient, or when measures proposed were seen as too reductive to reflect the complex work they undertook:

*‘… it just comes down to the patients, what’s best for them … using questionnaires to assess the patient’s mood … The GPs wanted me to do that for near enough every patient … that’s not useful because that score doesn’t tell us anything … people can fill the form out at different times of the day and different days of the week and get different results … ’*

(Site 2, LW01)

### Building a facilitative infrastructure

There were different experiences across sites of how far practices had prepared to integrate the link worker role. In some cases, link workers recalled being left to set up the social prescribing provision, which could feel overwhelming. Insufficient consideration of the infrastructure around the link worker role, when allocating money to PCNs, was criticised:

*‘… that’s another challenge that surgeries have because you’ve now got care coordinators and health coaches and all sorts and there’s only so many rooms. Doctors would take on these people and … the practice manager would tear their hair out trying to find space.’*

(Site 1, LW02)

*‘When the NHS Long Term Plan happened … it brought the PCNs into a very prominent position in terms of the social prescribing landscape. But at the same time, PCNs were being asked to do an awful lot in a very short space of time … For many of them you know, having to focus on quickly setting up, getting lots of different priorities sorted out, getting plans developed … it was a little bit messy, if I’m honest … ’*

(Site 6, HCP01)

Team building (or lack thereof) was mentioned across interviews. A failure to ask link workers to attend multidisciplinary team meetings was depicted as an oversight by some interviewees in learning about how these employees could assist patients. It was suggested that inviting link workers to coffee or lunch, providing them with an appropriate induction, and investing in their training were important:

*‘… my mandatory induction — which was terrible — they put down for me to shadow a nurse and the GP — well, it didn’t happen … When you start working in an organisation, you want to be received into that organisation and have things set up that make you feel welcome … ’*

(Site 4, LW01)

Data suggested that the infrastructure around a link worker should be reviewed regularly to respond to contextual changes. For example, some teams had expanded to bring in specialist link workers (to work with children and young people, or people with drug and alcohol issues). This reflected local needs and availability of resources — some of which came from external sources (for example, council funding). Such expansion could make it challenging for social prescribing leads to have the same shared ethos and values within the team as it grew:

*‘… you want to support them, but you can’t because by the very nature of the fact that you’ve got a big team, you can’t get around everybody like you might have done when it was smaller.’*

(Site 6, HCP04)

### CMOCs and programme theory (a continuum of belonging)

In line with a realist approach, we developed CMOCs (see Box 2) based on the four concepts described above to create a programme theory.

**Box 2.** CMOCs around link workers and their place within primary care

**Theme**	**Context**	**Mechanism**	**Outcome**
**Centralising or diffusing power**	If a PCN standardises social prescribing without consulting with those providing it	The skills and experience of LWs are not understood or known	So LWs are not employed in a manner that best serves the service/patients
	If an LW is accountable to a number of organisations	It can cause ambiguity	Leading to a lack of clear direction or support in the role
	The VCSE sector’s key role in the delivery of social prescribing is overlooked	This makes the VCSE sector feel put upon	Leading to disquiet and disengagement
**Forging an identity in general practice**	Primary care staff receive clear information about the LW role	They understand what these employees can do	So refer people who could benefit
	Experiences LWs bring to their role are understood by primary care staff	Increasing LWs’ legitimacy	Helping them to feel valued
	LWs presence in primary care and at team meetings	Means they become known by staff in the practice	Allowing a positive working relationship to be forged
	The LW role is made clear to patients	Who are then aware of what to expect	So patients are not disappointed with what is offered
**Demonstrating effect**	When LWs divert patients from seeing their GP for non-medical issues	Because they reduce inappropriate referrals to medical professionals	They are regarded as a useful addition to primary care
	When there is clear evidence that an LW is making a useful contribution to a practice	It gives credibility to the role	Meaning the service is used by stakeholders
	By developing a feedback loop	GPs get to hear how a patient has progressed with social prescribing	Which can increase the confidence referrers have in an LW and their skills
	When LWs are expected to collect data	They need to understand how it will benefit patients	Otherwise they will not be inclined to do so
	When success for LWs is judged by a PCN against indicators of throughput	It puts pressure on them to change how they work	Moving them away from providing person-centred care
**Building a facilitative infrastructure**	Consulting with key stakeholders about the delivery of social prescribing	Means that attention is paid to how it will fit into a practice	So social prescribing can be smoothly integrated into primary care
	Training and support are provided to LWs as required	Giving them confidence and skills	To effectively manage patients they are assisting
	When building connections between LWs and practice staff is seen as a two-way process	LWs and staff are facilitated to get to know each other	Which helps LWs to feel part of the primary care team
	Ongoing attention to how the LW role is experienced in primary care	Means the service can be responsive to fluctuating circumstances	So problems are picked up and addressed early on

*CMOCs = Context–Mechanism–Outcome Configurations. LW = link worker. PCN = primary care network. VCSE = voluntary, community, and social enterprise.*

**Figure 1.**
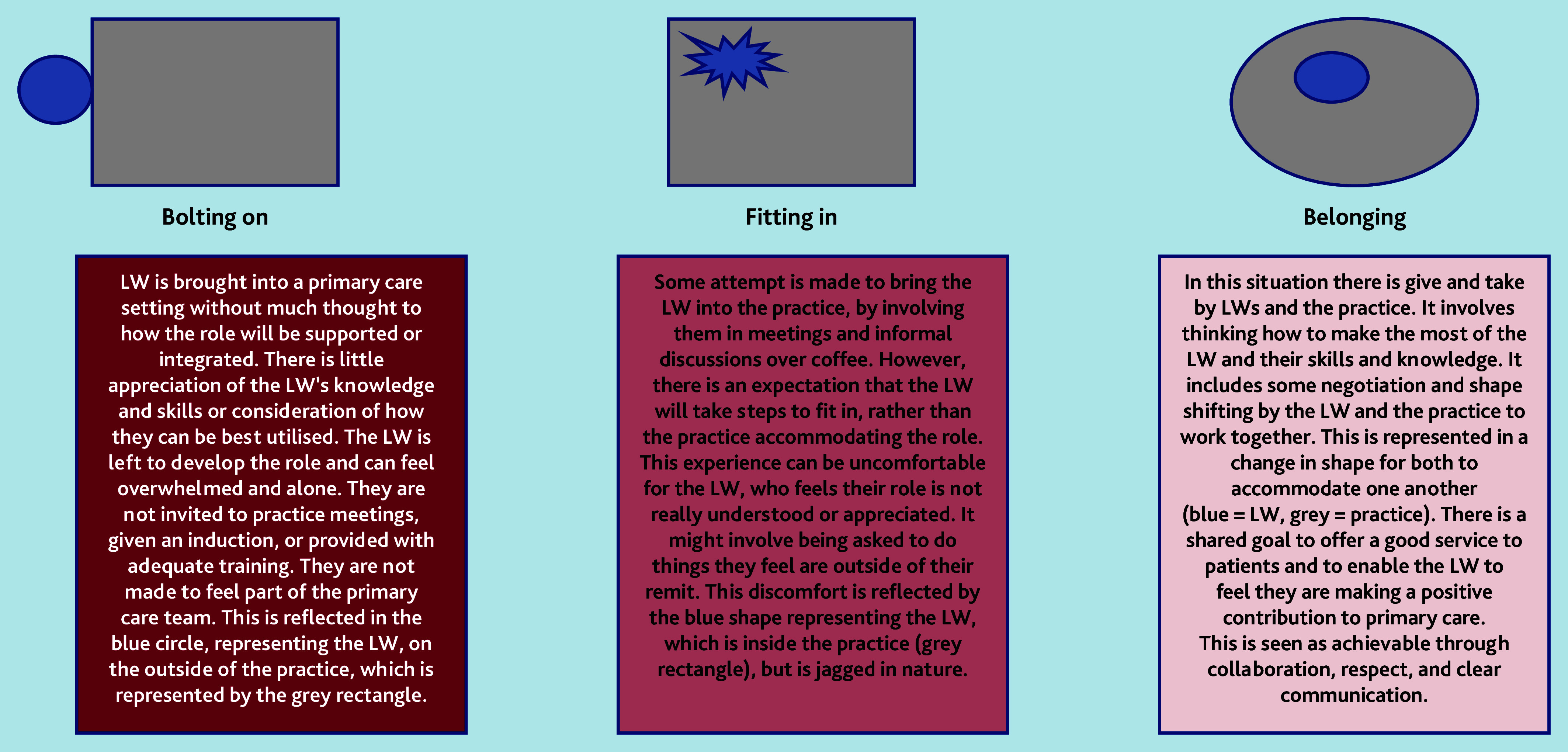
Different experiences around how LWs are embedded (or not) into primary care. From our analysis, it was clear that some LWs have been introduced in a way whereby they are bolted on or have opportunities to fit in, but rarely have space to feel truly integrated (as if they belong). LW = link worker.

As a team, we used these CMOCs to produce a continuum to explain how link workers experience being part of primary care. As reflected in Figure 1, at one end of the continuum, link workers are ‘bolted on’ — brought into primary care without consideration of how the role will work alongside existing provision, how their skills and knowledge will be used, or what additional support and training they require. This can leave link workers feeling isolated, potentially leading to a sense of overwhelm, whereby they consider leaving or do leave their job.

In a central position along the continuum is ‘fitting in’. This is when there is some attempt to bring link workers into the practice, but they are expected to conform to primary care and organisational targets. This can be uncomfortable as they feel their role is not really understood or appreciated, and they are unable to undertake the role as expected.

At the other end of the continuum is ‘belonging’, which is when the practice works with the link worker; some negotiation and potential adjustments may be required to offer the best support possible to patients, and to enable the link worker to feel they are making a positive contribution in primary care. The legitimacy of the link worker role is acknowledged by key actors in a practice and time given to link workers to make connections with the VCSE sector.

## Discussion

### Summary

Data highlighted it takes purposeful action to establish and sustain the link worker role in primary care. Minimal effort is likely to result in link workers being ‘bolted on’ to existing provision instead of feeling a sense of belonging that enables them to flourish, to have an impact, and to experience job satisfaction. The influence and legitimacy link workers have within primary care could be curtailed by their lack of qualification in social prescribing, inadequate systems for giving feedback on how patients were helped (or not), and poor interpersonal relationships developed with key stakeholders (for example, GPs).

### Strengths and limitations

We know that social prescribing programmes are diverse and context specific;^18^ our findings provide an insight from seven sites that were purposively selected to vary in terms of geography and area covered (affluent, deprived, coastal, and urban) on how link workers were employed and their tenure in the role. A range of participants took part. This allowed us to understand in depth the topic considered in this article. Through spending time with link workers at their place of work, we appreciated nuances associated with their role.

Reinterviewing participants enabled us to follow-up issues not considered when planning the study. Link workers involved were mainly White British and all were female, which is reflective of the gender balance associated with this role.^19^

### Comparison with existing literature

Existing literature on social prescribing has highlighted differing approaches to its delivery in primary care. For example, Calderón-Larrañaga and colleagues^14^ identified how link workers might go ‘above and beyond’ — doing what it takes to support patients (which may not be sustainable), or might adopt an uncritical approach, conforming to expected ways of practising social prescribing even if not in a patient’s best interests. Likewise, Griffith *et al*^10^ noted a dilemma faced by link workers in meeting referral and assessment targets against offering the intensive support some patients required.

Fallows^20^ wrote how current challenges in health care (for example, overprescribing medication, rising number of long-term conditions, and staff burnout) require a cultural shift in how patients’ needs are approached. This has contributed to a momentum for new ways of working — including greater integration between health and other sectors. Social prescribing is a means of directing people towards relevant VCSE services or resources best able to meet their non-medical needs.^21^ This can increase patient satisfaction and wellbeing.^22^ However, to access such support, patients must understand how social factors are affecting their health and be open and able to access appropriate support in the community. Previous research has highlighted how structural limitations, such as patients’ lack of existing capital — social, health, and cultural^23^ — alongside material or organisational constraints,^14^ can make this difficult.

Establishing an infrastructure that facilitates link workers to practise in a way that enables them to focus on personalised care is important. Our data, in line with other research,^24^^,^^25^ highlights the need for strong leadership, so people are clear about what is expected in the role and where to access support. Likewise, the VCSE sector’s key contribution to social prescribing cannot be overlooked;^12^^,^^26^^,^^27^ our data showed that this sector may struggle to establish its credibility in the eyes of some medical professionals and managers.

### Implications for practice

The continuum outlined in Figure 1 highlights aspects of the infrastructure where link workers need to be supported to embed this role in primary care. These aspects are detailed in Box 3.

**Box 3.** Aspects of infrastructure to consider to embed link workers into primary care

**Interpersonal:** The LW role is relational and relies on a strong, positive rapport between key stakeholders. Data suggested that LWs were expected to foster and sustain these interactions (for example, meeting with staff at a practice and making links with people working in the VCSE sector). This should be a two-way process, with practices taking steps to ensure that LWs feel welcomed and supported in primary care. The time LWs require to listen to and discover a patient’s story should also be respected. **Structural:** LWs require appropriate and adequate resources. This includes a vibrant VCSE sector to refer patients on to, clear information for key stakeholders about what they do, access to training and inductions, and appropriate space in which to see patients. **Procedural:** LWs must contend with several practical and bureaucratic factors because a range of stakeholders are key to social prescribing’s delivery. An understanding of the LW role could be enhanced by developing a feedback loop, so those involved in social prescribing, especially GPs as referrers, appreciate the contribution LWs make to patient care. Collecting and using data to improve a service ensures that the infrastructure continues to be appropriate and relevant, and can indicate changes required in its delivery and management. Any data collected need to be regarded as relevant by LWs to the assistance they give to patients (rather than a non-meaningful task). **Management:** LWs require clarity about who they can turn to for supervision about patient cases and their own wellbeing. This may be especially complex if LWs have line managers across primary care and the VCSE sector. It can also be an issue if those responsible for such supervision do not see it as a priority.

*LW = link worker. VCSE = voluntary, community, and social enterprise.*

In conclusion, a tension between fitting into an existing organisational setting dominated by medical practices, compared with feeling they belong, is something link workers may experience when providing social prescribing in primary care. Establishing an infrastructure that facilitates link workers to practise in a way that focuses on personalised, holistic care is important. This includes having appropriate supervision and training, and clarity around what is expected from their role in partnership with the wider primary care team. Steps that can be taken within a practice to foster a shared sense of belonging include assessing link workers’ impact using appropriate metrics, welcoming them to team meetings, providing them with inductions, producing clear information for patients about social prescribing, and allowing link workers time to develop connections with the VCSE sector.

## Data

Owing to the consent process for data collection with participants, no data can be shared except for quotations in reports, journal articles, and presentations. All data requests should be sent to the corresponding author.
